# Risk assessment and decision making about in-labour transfer from rural maternity care: a social judgment and signal detection analysis

**DOI:** 10.1186/1472-6947-12-122

**Published:** 2012-10-31

**Authors:** Helen Cheyne, Janet Tucker, Fiona Kane, Ashalatha Shetty, Sarah McLeod, Catherine Niven

**Affiliations:** 1 Nursing, Midwifery and Allied Health Professions Research Unit, University of Stirling, Stirling, UK; 2 School of Nursing Midwifery and Health, University of Stirling, Stirling, UK; 3 Division of Applied Health Sciences, University of Aberdeen, Aberdeen, UK; 4 Research Fellow, Nursing, Midwifery and Allied Health Professions Research Unit, University of Stirling, Stirling, UK; 5 Department of Obstetrics and Gynaecology, Aberdeen Maternity Hospital, NHS Grampian, Aberdeen, UK; 6 Mid Highland Community Health Partnership, Training & Practice Development Midwife, NHS Highland, Fort William, UK; 7 School of Nursing Midwifery and Health, University of Stirling, Stirling, UK

**Keywords:** Decision making, Risk assessment, Rural, Labor, Maternity care, Social judgment theory, Signal detection theory

## Abstract

**Background:**

The importance of respecting women’s wishes to give birth close to their local community is supported by policy in many developed countries. However, persistent concerns about the quality and safety of maternity care in rural communities have been expressed. Safe childbirth in rural communities depends on good risk assessment and decision making as to whether and when the transfer of a woman in labour to an obstetric led unit is required. This is a difficult decision. Wide variation in transfer rates between rural maternity units have been reported suggesting different decision making criteria may be involved; furthermore, rural midwives and family doctors report feeling isolated in making these decisions and that staff in urban centres do not understand the difficulties they face. In order to develop more evidence based decision making strategies greater understanding of the way in which maternity care providers currently make decisions is required. This study aimed to examine how midwives working in urban and rural settings and obstetricians make intrapartum transfer decisions, and describe sources of variation in decision making.

**Methods:**

The study was conducted in three stages. 1. 20 midwives and four obstetricians described factors influencing transfer decisions. 2. Vignettes depicting an intrapartum scenario were developed based on stage one data. 3. Vignettes were presented to 122 midwives and 12 obstetricians who were asked to assess the level of risk in each case and decide whether to transfer or not. Social judgment analysis was used to identify the factors and factor weights used in assessment. Signal detection analysis was used to identify participants’ ability to distinguish high and low risk cases and personal decision thresholds.

**Results:**

When reviewing the same case information in vignettes midwives in different settings and obstetricians made very similar risk assessments. Despite this, a wide range of transfer decisions were still made, suggesting that the main source of variation in decision making and transfer rates is not in the assessment but the personal decision thresholds of clinicians.

**Conclusions:**

Currently health care practice focuses on supporting or improving decision making through skills training and clinical guidelines. However, these methods alone are unlikely to be effective in improving consistency of decision making.

## Background

In many developed countries with significant rural populations the importance of supporting women’s choice to give birth close to her local community is recognized [1,2]. However, there are persistent concerns about the quality and safety of birth in rural areas [3,4].

Where local maternity services are not provided women living in remote areas may be required to travel long distances to urban centres to await birth [4,5]. These women may experience increased rates of induction of labour, emotional distress due to separation from family and community, and family financial hardship [5-7]. Further, risks to community sustainability and cultural safety have been highlighted in areas where local facilities for childbirth have been lost [4]. At the same time, even where community maternity facilities are available some women may opt to travel from their local area in order to receive obstetric led care which they perceive as being safer [8] and incidents relating to safety of maternity care in rural areas receive high profile media coverage. The paradox is that childbirth is viewed both as a normal life event and also as a time of increased risk and vulnerability. In the UK health policy supports the principle of choice of place of birth and birth close to local communities, within an integrated, multidisciplinary, maternity service [1,2]. However, there are continuing concerns about maintenance of skills of rural clinicians and about the safety of mothers and babies where unanticipated problems arise during labour in rural areas [9].

Community based maternity units in developed countries are typically supported by criteria which aim to stream women into low or high risk groupings during the antenatal period [1,10]. Characteristically, only low risk women will be booked to give birth in community maternity units where care may be provided by midwives, nurses or family medical practitioners. However, several studies have shown that general antenatal risk assessment is not effective in predicting birth outcome [11,12]. Approximately 36% of nulliparous and 10% of parous women deemed low risk are likely to develop unanticipated complications during labour requiring medical intervention [13]. In this situation in particular where local surgical back-up is not available, local clinicians must make the important decision whether to risk keeping the woman in local facilities or to transfer them to specialist obstetric services some distance away - a procedure which is in itself risky and distressing for mothers and babies and resource intensive for the maternity services [7,14].

The safety of rural maternity care is therefore dependent on the high quality of risk assessment and decision making of maternity care providers. There is evidence that this is a difficult decision. Wide variations in transfer rates between rural maternity units of similar size, geographical and demographic profile have been reported [11,15] suggesting different decision making criteria may be involved; furthermore, rural midwives and family doctors report feeling isolated in making these decisions and that staff in urban centres do not understand the difficulties they face [14,16,17]. A study of clinicians in rural Scotland found that they rated the importance of knowledge and experience in relation to risk assessment and the decision to transfer above specific clinical skills [16]. This report recommended the development and testing of ways to support reliable risk assessment and decision making in relation to remote and rural maternity services. However, despite their importance there has been little research on the way in which clinicians actually make case assessments and decisions or on what the possible sources of decision inconsistency may be. Greater understanding of these processes is required in order to develop evidence based decision making strategies for rural maternity care.

### Theoretical Basis: The General Assessment and Decision Making (GADM) model

The study was theoretically informed by The General Assessment and Decision Making (GADM) model [18] which provides a framework for understanding possible sources of variation in the judgment and decision making performance of clinicians. The model proposes that decision making results from three distinct elements:

A judgment: the clinician’s assessment of the level of risk facing a patient based on available cues or factors in the particular case

A decision: a choice between possible courses of action, for example, alternative treatment options, making a referral or even taking no action.

A decision threshold: linking the judgment and the decision.

**Figure 1:**
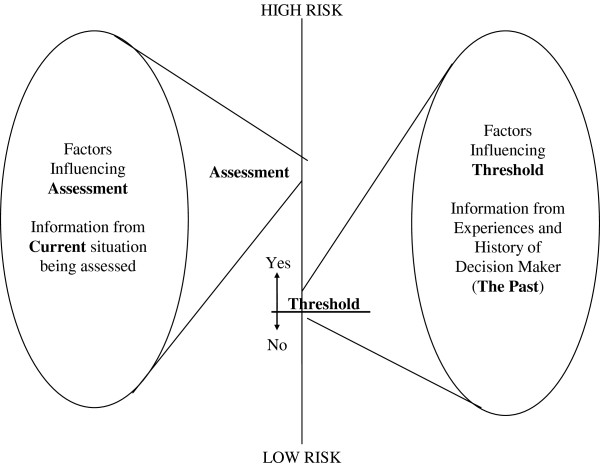
**The General Model for Assessment and Decision Making.** Reproduced with permission of Russell House Publishing.

These concepts are depicted in Figure 1. Good judgment depends on the clinician’s ability to distinguish between salient clinical or contextual factors in the case, and those which are less relevant. To make a good decision the clinician must accurately weigh up the risks and likelihood of possible decision outcomes based on the case assessment as well as knowledge drawn from their past experience and other sources of information. Assessments must often be made with incomplete information while outcomes of decisions are often uncertain. Given this complexity there is inevitably the chance of error [19].

The decision threshold is the link between the assessment and the decision. The threshold is like a line in the sand, when the assessed level of risk in the particular case crosses the threshold the decision maker will take action. If a clinician assesses a risk to be above their personal decision threshold a decision to act follows. If the risk is assessed to be below their threshold then they will withhold action. The decision threshold of risk which an individual is likely to tolerate is based on the utility, or value that they place on the consequences of each possible decision outcome and their belief about how likely it is to occur [18]. This is influenced by the past experience of the individual decision maker, personal or vicarious, including relevant emotional events. Individuals have different personal experiences and therefore different decision thresholds, which do not alter on a case by case basis.

### Applying GADM to the decision to transfer during labour

The GADM model suggests that the decision to transfer will stem from the clinician’s assessment regarding the level of risk in a case and their personal threshold of tolerable risk. Table 1 illustrates the complexity of this task in the context of the decision to transfer a woman during labour. There are four possible decision outcomes, (a) and (d) are “correct” decisions. There are potentially negative consequences associated with each of options (a), (b) and (c), for example, even a correct decision to transfer may require a lengthy journey by road or air for a woman in labour. However, it may not always be possible to discriminate, even retrospectively, between options (a) and (b). In this situation a good decision maker is one who is able to discriminate between women who do need to be transferred and those who do not, aiming to maximize “hits” that is true positives and true negatives and minimize “misses” false positives and in particular, false negatives (c) which may have severe consequences for the wellbeing of mother and baby.

**Table 1:** Outcomes for the decision to transfer

	**“True” situation**	
	**Woman should have been transferred**	**Woman should not have been transferred**
**Midwife decides to transfer**	(a). correct decision to transfer (true positive - HIT)	(b). wrong decision to transfer (false positive - MISS )
**Midwife decides not to transfer**	(c). wrong decision not to transfer (false negative - MISS)	(d). correct decision not to transfer (true negative - HIT)

In highlighting the link between the assessment and the decision the GADM model identifies two potential sources of inconsistency between clinicians. Clinicians may have the same decision threshold but differ in their assessment of the level of risk in a case. Alternatively they may agree about the assessed level of risk and yet have different decision thresholds. In both these situations one clinician will act while the other will not although the source of disagreement is different [20].

Given the clear importance of maximizing appropriate and safe decision making regarding the decision to transfer a woman in labour it is important to gain understanding about both the assessment of risk and the decision threshold. This study aimed to examine how midwives and obstetricians make intrapartum transfer decisions and to describe possible sources of variation in decision making. In particular we sought to address the following research questions:

1. What case and contextual factors influence intrapartum transfer decisions?

2. What are the relative contributions of these factors to case assessments?

3. Do these factors and their relative contribution to assessments vary between midwives and obstetricians and between different types of midwifeled maternity unit?

4. To what degree can clinicians distinguish higher risk cases from lower risk cases and the overall level of risk required (across the case) before the decision to transfer is made?

5. Do thresholds for decision making vary significantly between midwives and obstetricians and between different types of midwifery unit?

## Methods

The methodological approach for the study was informed by both Social Judgment Theory [21,22] (Table 2) and Signal Detection Theory [22-24] (Table 3). Both of these processes use vignettes in order to explore decision making. Although the use of hypothetical scenarios cannot replicate the stress of decisions made in the real life clinical situation, presentation of the same case information to each study participant overcomes the problem that no two clinical situations are ever the same, and allows the development of predictive models of the relationship between case factors and decisions [25]. This approach has previously been used in the study of child protection and judicial decision making [20,26].

**Table 2:** Social judgment theory

**Theory**	**Design**
Social Judgment Theory (SJT) studies the relationship between the case factors, or cues, and the assessments and decisions which are actually made (research questions 2 and 3). It is based on the notion that people must make assessments (judgments) based on the information available to them and that this is often incomplete or ambiguous. From available information they make inferences about the “true” situation. Exploring the way in which particular information factors are used provides evidence about judgment accuracy and variability between the judgments people make.	In SJT vignettes are used in which the same case information (factors) is presented to each participant. Using SJT vignettes may be narrative or graphical in form although where SJT and SDT are combined graphical vignettes are used. Factors included in vignettes are typically elicited from people who are experienced in making the assessment to be studied through interviews from which relevant factors are abstracted. The same factors are included in each vignette however, the level or weight of each factor is randomly varied across the vignettes, to provide a range of risk levels (0-100) for each (0 represents no concern and 100 the highest possible concern). Selection of relevant factors and realistic factor weights is achieved by developing and piloting vignettes with appropriate experts, this ensures that although the vignette format may be abstract the factors and weights are recognisable (ecological validity) as those which could occur in real life.
**Administration**	**Analysis**
Study participants are asked to rate the overall level of risk (0-100) for each vignette and decide whether they would act or not act. Vignette tasks using SJT can become very large. Between five and 10 vignettes are required for each factor included to allow for the regression analysis; therefore no more than 10 factors are usually included in each vignette so that the overall task does not become onerous for participants.	Judgment analysis identifies the relative contribution of each factor and weight of each factor to the overall assessment of risk in the case, and the decision to act (i.e. what factors are used and how they are used). Linear regression is used to model the continuous judgment about level of risk in each vignette (0-100) and logistic regression to model the dichotomous choice (act or no action). Varying the factor information presented to study participants across vignettes, allows the responsiveness of clinicians to differing factor information to be established, this is fit and is measured by the multiple correlation coefficient (values above 0.6 are expected). Comparison of scores between participants identifies variability within and between clinician’s judgments. Mean scores between individuals and groups are compared using t-test for independent samples. Repeat cases are used to identify judgment consistency.

**Table 3:** Signal detection theory

**Theory**	**Design**
SDT uses vignettes in which a forced dichotomous choice task is used to test the participant’s ability to detect a “signal” against a background of “noise”. For each vignette the participant decides whether the signal is present or absent. The signal can be any event or state that the person has to judge and the noise is the additional information which is presented. When used in decision making research SDT is based on the notion that a decision maker must have the ability to detect the need to take action i.e. to discriminate between high and low levels of risk in a case, and have a personal decision threshold which determines the level of risk they will accept before deciding to take action. SDT assumes that on average, skilled people are more likely to take action where there are higher levels of risk than in low level risk cases. At either end of the risk spectrum (high risk to low risk) the majority of skilled decision makers would agree, but there is a “grey area” where cases in which there is a need to take action, and those where no action is required overlap. The point at which the decision to act is made indicates the individual decision threshold.	Information about the level of risk comes from the case assessment and is case specific. The personal decision threshold is based on belief about the likelihood and utility for possible outcomes and is relatively fixed across cases. For example, a clinician who believes that failure to progress in labour is likely, or that it will result in very negative consequences will require a lower level of risk before taking action than the clinician who believes it is unlikely to happen or have only minor consequences.
	SDT uses vignettes which are developed as for SJT described in Table 2. However, to allow for the SDT analysis vignettes are specifically selected for inclusion in the task so that 50% have an average of a high level of overall risk across the factors (e.g. 60 out of 100), and are designated to be signal or “should take action” cases. 50% are selected to have a low average amount (e.g. 40 out of 100), of risk across the case factors and are designated as no signal or “should not take action” cases.
**Administration**	**Analysis**
Participants are asked to decide for each case whether they would take action or no action	Using this method, for each vignette a decision to act could be a true positive or false positive. The decision making performance of participants is captured by their true positive and false positive rates. These scores are turned into two indices of performance, ability to discriminate “should act” cases from “should not act” cases and the decision threshold (willingness to act) which is determined by the level of risk required in the case, before the decision to act was made. These analytic methods yield standard errors for the relative weights and thresholds and this allows comparisons between individual midwives and obstetricians using Z-tests. Ability has a minimum of zero when the participant has no ability. Willingness has a negative value when the participant has a greater willingness to act.

The study was conducted in three stages.

**Stage 1:** The identification of factors that influence risk assessments by midwives and obstetricians (research question 1).

**Stage 2**: The development of case vignettes reflecting the factors identified in stage 1.

**Stage 3**: The identification of the relative contribution of factors to the assessment and the decision and comparison of decision threshold levels through completion of the vignette task created in stage 2 (research questions 2–5).

### Setting and sample

The study was set in Scotland which has a population of just over five million people, one third living in rural areas. With a relatively small land mass (30,414 square miles) accessibility and travel time define rurality. Rural is defined as settlements with a population less than 3000; remote rural areas are defined as those with greater than 30 minutes’ drive time to the nearest settlement with a population greater than 10,000 [27]. Maternity care provision comprises 17 urban consultant led obstetric units, five with alongside midwife led units (MLU) and 22 stand alone community midwife led units (CMLU). In all locations midwives are the lead care providers for normal healthy women, making referral to the appropriate medical personnel as required.

Stratified random sampling was used. A stratified sampling frame was constructed classifying all CMLUs and MLUs in Scotland by type of maternity unit [1], “far/near” based on travel time to acute service provision above or below the mean, and “high /low” reported intrapartum transfer rate based on rates above or below the mean reported rate. This allowed stratification and subsequent analysis by these factors. Where possible, three maternity units were randomly selected from each cell of the sampling frame. Midwives from these units, who provided intrapartum care, were eligible for study participation. Two thirds of the sample was drawn from CMLU and one third from MLU in order to maintain the rural focus. Two island CMLU were excluded as local medical staff routinely undertake labour interventions including induction of labour and caesarean section. Of the 17 consultant led obstetric units in Scotland four provide the main service for intrapartum transfers from rural units. Consultant obstetricians with key responsibility for the labour ward, maternity team working and clinical governance were purposively selected from these four maternity units.

### Recruitment and consent

For both stages a ratio of midwives were randomly selected from each unit’s establishment lists, by local link midwifery managers using a random number sequence generated by the research team. The link midwife first arranged the establishment list alphabetically then paired it with the random number sequence; this identified the midwives to approach for study participation and the order in which they should be approached. These midwives were each given a pack containing study information and an invitation to participate. Midwives who wished to do so returned a study consent form and contact sheet to the research team. This process was repeated until the target sample was achieved. Midwives participated outwith their normal service commitment and were reimbursed for time and inconvenience. Obstetricians were identified via the local Clinical Director and were sent study information and invited to participate.

### Ethics and research governance

The study received ethical approval from MREC A Scotland (07/MRE00/114) and Research and Development offices within each participating area.

### Stage one: Identification of factors contributing to in-labour transfer decisions

Individual interviews were conducted using Critical Incident Technique [28] to identify contributory factors for the decision to transfer. Midwives and obstetricians were approached as described above. Our target sample for the stage one interviews was 21 midwives and four obstetricians. It was anticipated that this sample would generate around 105–126 critical incidents (100 critical incidents are considered adequate for generalisation [29]). Twenty midwives (53% consent) and four obstetricians (80% consent) participated. Midwives were asked to recall specific cases which had required challenging judgements about the appropriate level of care in labour, in which a range of decisions were made (i.e. transfer or not) and to report what factors influenced their decisions (Table 4). Obstetricians were asked to recall similar cases from their perspective as a receiving clinician. These are critical incidents; the details of such cases are not easily forgotten. The technique uses ‘what’ type of questions rather than ‘why’, this lessens the possibility of self justifying bias. Interviews were audio recorded, transcribed and analysed using manifest content analysis [30] specifically identifying factors, clinical and contextual that led to the decision to transfer as well as the frequency of factors across the cases. This method was used to facilitate subsequent development of realistic vignettes. Initially five interview transcripts were analysed by four members of the research team (FK, HC, JT LD). Findings were discussed and an overall framework for the remainder of the analysis was agreed.

**Table 4:** Interview guide for critical incident technique

**Questions to elicit specific cases (Part A) and questions asked about each case (Part B)**	
**Part A**	**Part B**
1. Think of a transfer case where it was clear that the woman should have been transferred.	1. What pieces of information, that is, cues or factors, did you use to make the decision to transfer?
2. Think of a case where it was clear that the woman should not have been transferred.	2. What were the factors in the case that most strongly led to the decision you made?
3. Think of a ‘grey area’ case where it was unclear whether the woman should or shouldn’t have been transferred.	3. What other pieces of information influenced your decision? What aspects made the case clear/typical/similar/difficult?
4. Think of a ‘typical’ or ‘common’ decision to transfer case.	4. What particular aspects of this factor were important?
5. Think of a case where you decided to transfer but thought you’d made an error.	
6. Think of a case where you didn’t decide to transfer but thought you’d made an error.	

#### Stage one findings

Participants described between five and 12 cases eliciting 160 cases overall. The obstetricians tended to describe information amalgamated from typical cases while midwives were more focused on specific cases. Analysis identified three main categories and associated factors (Table 5). These related to 1) The mother- clinical characteristics of mother and baby, physical and psychosocial factors and family 2) The context - local geography and transport assessment, judged time available for transfer and 3) The characteristics of the service - use of guidelines, impact for the midwife, workload for the midwives unit, attitude and capacity of the receiving unit.

**Table 5:** Categories and case factors elicited from critical incident technique interviews (frequency in the 160 cases described)

**Category**	**Case factors**	
Mother	Fetus/ Baby	pre-birth condition (45); fetal heart rate (63); meconium (30) fetal size (11) post-birth condition (19); Strep B (5) other (9)
	Clinical (mother)	blood loss – pre/post birth (44); blood pressure (38); obstetric history (32); pain/analgesia (47); progress in labour (123); other (32)
	Physical	parity (116); general condition (62); gestation; (41); age (8); BMI (11), other (39)
	Psychosocial	attitude (30); coping (33); preference (53); planned place of birth (15)
	Family	father’s attitude / state (10); logistic problems (3); other children (3)
Context	Judged time available (23)	
	Logistic factors	transfer time (69), geography (10) weather (9) time of day (42), transfer problems (36)
Service	Guidelines (48)	
	Midwifery	awareness of impact on local area (3); decision making (16) past experience (46); fear of litigation (8); psychosocial (135)
	Midwife led Unit	staff cover (31); maintaining viability of unit/costs (9)
	Receiving Unit	attitude / communication (32); capacity / resources (10); medicalisation (4); opinion of others (16)

In most cases participants reported that clinical factors most strongly led to the decision to transfer. Concern about progress in labour was the most commonly described factor.

"‘she’d been three hours (cervix) fully dilated, having ruptured her membranes prior to that and still no visible presenting part despite the fact that she had been actively pushing’ (midwife 8)."

"‘I knew that labour was slow and it was getting to the stage that it was becoming slower because of maternal exhaustion, she was becoming ketotic’ (midwife 17)."

However, a wide range of additional physical, social and contextual factors were also reported to influence the decision. For example, the midwives considered the woman’s parity (was this a first or subsequent birth?), her preferences, and the logistical implications for the family of transfer.

"‘for her husband it was difficult because they didn’t allow him to stay (in the hospital) and they didn’t have enough money for him to stay in lodgings in Glasgow’ (midwife 4)."

They were also aware of the wider implications of their decision making within their local community both in relation to the impact on maternity service provision and concern about the opinions of others.

"‘we’re very aware of centralisation. We can provide great care but we’re very conscious that we need to keep our numbers up’ (midwife 1)."

"‘I was more worried about what folk would think about my decisions, I was just a bit more nervous’ (midwife 5)"

### Stage 2: Development of vignettes

Data from stage one was used to develop vignettes as described in Tables 2 and 3. The same overarching clinical scenario was fixed for each vignette and described at the start of the vignette task. This was a primiparous woman in active labour at term, suitable for midwife led care on admission and where concern has subsequently developed about progress in labour. Progress in labour was chosen as this was the most common reason given for transfer and as the diagnosis is rarely clear-cut, with a number of variable clinical factors coming in to interplay. Characteristics such as gestation, general health and clinical observations were normal and were also fixed for each vignette. Ten factors were included in the vignettes five were clinical, relating to aspects of progress in labour, five were non-clinical relating to context and service issues (Table 6). The same factors were included in each vignette but the risk level for each was randomly varied between vignettes (0 representing no risk and 100 highest possible risk). SJT analysis requires between 5–10 vignettes per factor (Table 2) therefore 72 vignettes were included in the task. For the SDT (Table 3) 50% of the vignettes were designated as ‘should transfer’ cases and 50% ‘should not transfer’ cases. Eighteen cases were repeated to assess intra-rater consistency.

**Table 6:** Factors included in the vignettes

**Factor**	**Aspects**
*Clinical*	
Mother	Physical condition of the mother-coping, hydration, vital signs, demeanour
Descent	Descent and position of the fetal head
Cervix	Condition of the cervix **–** dilatation, effacement application
Contractions	Characteristics of the contractions-strength, frequency, regularity
Fetus	Condition of the fetus- liquor and etal heart
*Non - clinical*	
Agreement	Level of agreement between mother and midwife about place of birth –preference, attitude to transfer, expectations
Partner	Attitude of birth partner– emotional, support of partner, knowledge and expectations
Consultant Led Unit (CLU)	Attitude of receiving staff to midwife making the phone call and to birth unit staff
Midwife Led Unit (MLU & CMLU)	Characteristics of the birth unit – workload, support, time of day, tiredness
Transfer	Transfer issues- availability of care, availability and type of transport , weather

**Figure 2:**
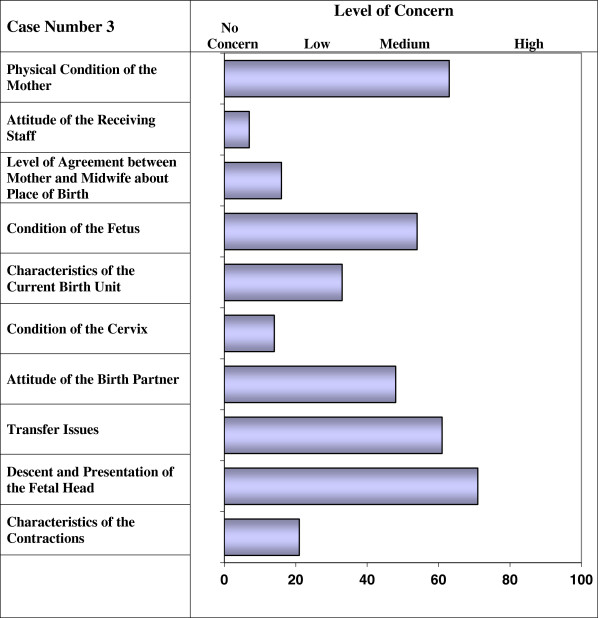
Vignette Example.

The vignettes were reviewed to ensure that factors were compatible with each other and realistic. Based on data from stage one and in consultation with clinical midwives, a training manual and booklet of practice vignettes were developed in which the factors were defined and examples of low, medium and high levels of risk were described. The vignettes and training materials were extensively piloted with midwives before use in stage two. Initial piloting identified that a risk level of 60–100 across the vignettes was required to clearly distinguish “should transfer cases” therefore this level was chosen for the main vignette task. Some uncertainty was also identified about the use of the term “risk” which has several meanings within clinical practice. In the UK midwives often associate the term with clinical risk management. Therefore the term “level of risk” was replaced with “level of concern” for each of the factors (vignette example is shown in Figure 2).

### Stage 3: Completion of the vignette task

The target sample for undertaking the vignette task at stage three was 120 midwives and 12 obstetricians. For the correlational (regression) analysis this sample size has the statistical power (power = 0.8 at α 0.05 to detect a medium effect size (correlations of the order of 0.3) between decision making performance and individual difference variables. For the analysis of variance this sample size has the statistical power (power = 0.8 at α 0.05 to detect a medium effect size [31].

For each vignette participants were asked to rate how suitable the woman was to remain in midwife led care (0-100mm) and to decide whether or not to transfer the woman to obstetric led care. Vignettes and factors were presented in random order to avoid practice effects.

Analyses were conducted as described for SJT in Table 2 and SDT in Table 3. Three aspects of the assessment were considered; these were variation within and between participants, “fit” which indicates participant’s responsiveness to the variation in cue or factor weights across cases, and the relative importance (weights) for each of the case factors in making the risk assessments and the decision to transfer. Variation in the decision threshold was assessed using SDT (Table 3) considering participants ability to discriminate “should transfer” from “should not transfer” cases and the overall level of risk required (across the case) before the decision to transfer was made.

## Results

Recruitment by stratification is shown on Table 7. One hundred and twenty-two midwives (56% consent) and 12 obstetricians (100% consent) participated. The characteristics of midwives, by type and level of unit are shown on Table 8. There was no significant difference between units in aspects of midwives’ clinical experience. Midwives were asked to estimate the travel time required for in labour transfer from their unit to acute service provision. This ranged from five to 240 minutes and corresponded to unit type.

**Table 7:** Recruitment of midwives by stratification level for interviews and vignettes

**Type**	**High transfer rate/ far travel distance to acute care**	**High transfer rate /Near travel distance to acute care**	**Low transfer rate /Far travel distance to acute care**	**Low transfer rate /Near travel distance to acute care**
CMLU units available	3 Units	6 Units	6 Units	5 Units
Eligible midwives	n = 22	n = 109	n = 46	n = 41
Interviews	n = 3 (6)	n = 4 (11)	n = 3 (3)	n = 3 (4)
Vignettes	n = 19 (24)	n = 21 (51)	n = 21 (34)	n = 21 (30)
(number approached)				
MLU units available	Not valid	4 Units	Not valid	1 Unit
Eligible Midwives		n = 204		n = 24
Interviews		n = 6 (12)		n = 1 (2)
Vignettes		n = 21 (60)		n = 19 (20)

CMLU (total n = 20) community midwife led units.

MLU (total n = 5) midwife led unit alongside a consultant led maternity unit.

**Table 8:** Participants’ years of experience and perceived travel time to acute care

**Item**	**Stand alone CMU**				**MLU alongside CLU**		**Obstetrician**
	Distant		Near				
	High T/R	Low T/R	High T/R	Low T/R	High T/R	Low T/R	n = 12
	n = 19	n = 21	n = 21	n = 21	n = 21	n = 19	
Years: mean (SD)							
Qualified	22.8 (12.5)	18.2 (7.6)	22.0 (8.2)	22.0 (8.4)	22.1 (7.3)	19.8 (9.7)	23.2 (9.1)
In practice	18.8 (10.2)	16.6 (7.4)	18.9 (8.4)	19.8 (8.1)	21.6 (7.3)	17.3 (9.7)	-
In midwife led care	11.7 (6.4)	9.1 (4.7)	7.6 (6.6)	7.4 (6.4)	10.6 (3.7)	11.4 (7.3)	-
Mean perceived travel time to acute care in minutes (range)	141 (75- 210)	159 (120- 240)	56 (30- 120)	96 (45–210)	20 (5–30)	14 (0–30)	-

T/R- transfer rate.

### The assessment

Participant’s assessments about whether cases remained suitable for continuing midwife led care across vignettes were not significantly different between midwives from units stratified by distance to acute care, by high /low transfer rate or between midwives and obstetricians (Table 9). From the 18 repeated vignettes the correlation between participants’ assessments for the first and the repeated presentations measured consistency (test-retest reliability) of judgments. There were no differences between the groups. The mean consistency was 0.59 indicating only a moderate degree of consistency. The overall mean fit was high (0.81) indicating that participants were able to ‘read’ the case factors and integrate the information in similar ways across the vignettes and there were no significant differences between the groups.

**Table 9:** The assessment

**Item**	**Stand alone CMU**				**MLU alongside CLU**		**Obstetrician**
	Distant		Near				
	High T/R	Low T/R	High T/R	Low T/R	High T/R	Low T/R	n = 12
	n = 19	n = 21	n = 21	n = 21	n = 21	n = 19	
Mean suitability for Midwife led care (SD)	39.1 (11.1)	39.0 (8.5)	41.6 (8.2)	44.8 (12.3)	41.1 (8.2)	43.0 (10.9)	44.6 (16.3)
Consistency (SD)	0.55 (0.22)	0.59 (0.22)	0.66 (0.14)	0.59 (0.22)	0.59 (0.20)	0.59 (0.23)	0.55 (0.28)
Fit (SD)	0.80 (0.06)	0.80 (0.05)	0.82 (0.05)	0.80 (0.06)	0.81 (0.05)	0.82 (0.04)	0.80 (0.06)
Variance for non-clinical factors % (range)	3 (0.8- 12)	4 (0.9- 14)	3 (0.9- 9)	4 (1–9)	3 (1–12)	3 (1–9)	4 (0.4 - 8)

**Figure 3:**
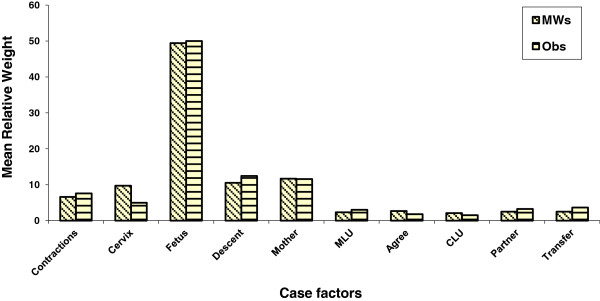
Relative weights for case factors.

The relative contribution of each case factor to the judgment is shown in Figure 3. There were no significant differences between groups. Clinical factors dominated with the condition of the fetus being the most important factor. The relative contribution, to the judgment, of non-clinical factors is shown in Table 9. Overall, non-clinical factors accounted for only around 4% of variance in the judgment with a range of <1% to 14%.

### The decision threshold

The groups did not differ significantly in their ability to discriminate between ‘should transfer’ and ‘should not transfer’ cases (Table 10). However, there was a significant difference in decision threshold identified by the proportion of transfer decisions and the willingness to transfer. Midwives working in units which were distant from acute service provision transferred significantly more cases (60% v 46% t (120) = 3.9; p = 0.0001) and had a lower threshold for transfer (−0.24 v 0.34 t(120) = 3.61; p = 0.0001) than midwives in units near or alongside acute service provision. Obstetricians did not differ significantly from midwives in measures of threshold placement. However, within all groups the range of transfer decisions was very wide demonstrating considerable variation in decision making practice.

**Table 10:** The decision

**Item**	**Stand alone CMU**				**MLU alongside CLU**		**Obstetrician**
	Distant		Near				
	High T/R	Low T/R	High T/R	Low T/R	High T/R	Low T/R	n = 12
	n = 19	n = 21	n = 21	n = 21	n = 21	n = 19	
Ability (range)	1.25	1.14	1.15	1.28	1.26	1.37	1.13
	(0.56-1.89)	(0.08-2.05)	(0.40-2.42)	(0.31-1.94)	(0.69-2.27)	(0.57-2.48)	(0.28-1.84)
Decision to transfer % cases (range)	59 (29–88)	60 (25–93)	51 (13–72)	43 (7–78)	46 (25–65)	45 (25–85)	53 (1–96)
Willingness to Transfer	−0.30	−0.19	0.09	0.47	0.32	0.49	−0.17
	(−2.19 to 0.96)	(−0.83 to 1.83)	(−1.7 to 2.19)	(−0.93 to 1.83)	(−0.47 to 2.42)	(−1.04 to2.42)	(−1.83 to 1.25)

## Discussion

Many healthcare judgments are made in situations of uncertainty where assessments and decisions are based on information that is incomplete, made under time pressure and in an emotional atmosphere. This study examined one such situation, the decision to transfer a woman in labour from a community maternity unit to a specialist obstetric unit; a decision which is central to the provision of high quality, safe maternity care in rural areas.

The study found that when presented with the same case factors participants made similar judgments about women’s suitability to remain in midwife led care. In the first stage of the study midwives and obstetricians described a wide range of clinical and contextual factors which they reported taking into account in deciding whether to transfer a woman in labour to specialist obstetric facilities, including the woman’s preference for place of birth, impact on the family, workload, the attitude of the receiving unit, and travel time. However, subsequent analysis of the vignette task using SJT found that clinical factors dominated the assessment, with one key factor, concern over wellbeing of the fetus clearly paramount; contextual issues appeared to play little part. Other studies have reported a perceived lack of understanding between midwives working in different settings [14,16,17], and this issue was raised in stage one. However, the vignette analysis found that, regardless of professional group or setting, clinicians made similar case assessments, using the same case factors and weighing them similarly.

Despite making very similar case assessments, there were significant differences in the decisions that were made. Distance was an influencing factor, midwives working in units which were more distant from acute service provision made significantly more decisions to transfer and were more willing to transfer cases than midwives working in near or alongside midwife led units, or obstetricians. More surprising, was the wide range of transfer decisions that were made within all groups. For example, while one midwife (from a distant unit) decided to transfer only 25% percent of cases another midwife, within the same group, would have transferred 93%. The largest differences were found in the obstetrician’s group where the range was 1-96% of cases. This may be partly explained by the relative unfamiliarity of the task for the obstetricians, as within the UK they invariably receive transferred cases and will not have personal experience of making the decision to transfer from a rural maternity unit. From our findings it appears that variations in transfer decision making lie not in the clinicians risk assessments but in their risk tolerance and personal decision thresholds, and that this is exacerbated by distance from acute care.

The quality and safety of rural maternity care is an issue of continuing debate in particular in developed counties with extensive remote and rural areas, for example, Canada and Australia. However, within geographically smaller countries such as the UK the principle issues of concern are the same, despite the smaller distances involved. Much of the research on rural maternity care has focussed on clinical and economic outcomes [3,14,32-34] and on the clinical skills, and competence of healthcare providers [9,16]. As a result there has been a focus on provision of guidelines and training strategies targeting maintenance of skills and improvement of clinical assessments. Clinical knowledge and competence are clearly essential aspects of high quality healthcare however, the findings of the current study suggest that clinicians across a range of settings have the ability to make good clinical asessments and that the clinician’s personal decision threshold is more influential.

Decision thresholds are determined by an individual’s values and their utilities for the consequences of their decisions. Studies reporting the experiences of rural clinicians highlight why this may be an issue of particular relevance for rural maternity care. Practitioners have reported feelings of personal responsibility for sustainability of local services, and for the consequencies of poor clinical outcomes [16,17]. These experiences are exacerbated by the visability of healthcare workers within local communities and by feelings of isolation and lack of understanding and support from colleagues working in urban referral centers [14,16,17,33]. Maternity care practitioners are acutely aware of the risks and uncertainties inherent in their judgments and decisions, and of the high stake, long term consequences both for themselves and the communities they serve.

This is the first study which has focussed specifically on the judgment and decision making performance of rural healthcare providers, using a model informed by decision making theory. It provides an explanation for the wide range of decisions made against a background of similar clinical assessment. Key to the model is the notion that the factors influencing the assessment of a case are different to those influencing the placement of the decision threshold. Overall, clinicians appear to take into account the same pieces of case information and combine these data in similar ways; it appears that the “scales” used in making case assessments are similar, however, their decision cut off points are different. If a clinician has a low decision threshold then they would take action (transfer) even if they assessed a case to have low risk. Conversely, if the threshold was high, then they would take action only if the risk assessment is high. Consequently, even if two clinicians agree on the amount of risk in a case, they may disagree about the course of action because their tolerance for acceptable risk differs.

These findings have important implications for clinical practice. In some situations the decision task may be relatively clear cut, objective diagnostic measures may be available for the assessment along with strong, evidence based guidelines for clinical management. An example would be hypertension in pregnancy where a blood pressure recording above a specific threshold will trigger a medical referral in the majority of cases. However, in many clinical situations uncertainty characterises both the assessment and the decision. In these cases there will always be the need for clinicians to exercise professional judgment, increasing the likelihood of variation, yet consistency is considered to be one of the key markers of quality healthcare. While consistency is not a guarantee of good decision making (clinicians could be consistently wrong) inconsistent decisions must, at best, be correct only some of the time. The response to inconsistency or apparent error in clinical decision making is often to introduce guidelines or to undertake case reviews. However, where clinicians do not differ, or do not differ by very much in their case assessments, retrospectively reviewing cases, or trying to improve consistency of clinical assessment by introducing guidelines (in particular guidelines based chiefly on professional consensus) is unlikely to identify the source of disagreement or increase consistency of decision making.

There has been little research on possible means of adjusting decision thresholds and more applied research in this area is required. Decision thresholds are affected by experiences both personal and vicarious; thresholds may change gradually over time or shift rapidly in response to traumatic events. Such events may remain vivid in the memory for long periods and may even be passed down in the ‘folk memory’ of a hospital. While it is not easy for people to choose to change the values they attach to consequences which have been shaped by past experience and history, understanding the sources of decision conflict and provision of peer support may provide the opportunity to bring decisions closer together. The reverse is also likely to be true where punitive responses to clinical error may have the effect of lowering decisional threshold rather than improving clinical assessments.

### Limitations

The use of vignettes cannot fully replicate the real life clinical situation where judgments and decisions are rarely made in isolation. However it allows the presentation of same case factors to all participants, a situation which cannot be replicated in real life. The ecological validity of the vignettes was maximised by extracting data from a large number of cases described by clinicians and extensive piloting of vignettes and training materials. Further, although the vignettes were abstract in form the decision task was very familiar to the midwife participants, less so for the obstetricians. The rigorous sampling method used means that the study findings are likely to be representative of midwives providing rural maternity care across Scotland although this is less likely to be the case for the obstetricians where purposive sampling was used and smaller numbers were involved. Nevertheless, the study involved a large clinically relevant sample, this contrasts with many decision making studies which are often characterised by small, student samples.

## Conclusions

Clinical assessment and decision making are core activities in all healthcare settings. This study has demonstrated that the midwives working in rural maternity care and receiving obstetricians have good case assessment skills, focusing on salient clinical factors and placing less emphasis on contextual “noise”. This is reassuring for the provision of safe rural maternity care, and will be of benefit in developing future guidelines and training for rural practitioners. However, the study also found considerable inconsistency in decisions made and this may be more resistant to change through provision of protocols or clinical guidance. It appears that understanding the importance of personal values that underpin clinical decision making is a necessary step to improving the quality of rural healthcare. While individual clinicians may benefit from having insight into a source of decisional conflict between colleagues, it also may be important for policy makers and rural communities to consider what a desirable decision threshold for in-labour transfer would be. Further research is required to identify values and preferences of service users, rural communities and policy makers in the provision of safe rural maternity care.

## Misc

Len Dalgleish deceased

## Competing interests

The authors declare that they have no competing interests.

## Author’s information

HC is a midwife by professional background. The study uses a decision making research method developed by LD, not previously applied to a health care setting. LD died following completion of the study and final report, but before writing of this paper began.

## Authors’ contributions

HC conceived of the study, participated in its design, coordinated the study and wrote the manuscript. LD developed the study method, participated in the design of the study, performed the statistical analysis and wrote the study final report with HC. JT participated in the design of the study, analysis of data and helped to draft and revise the manuscript. FK organised the study, collected the data, contributed to the analysis and the drafting of the manuscript. AS contributed to the study design and the drafting of the manuscript, SMcL contributed to the study design and the drafting of the manuscript, CN contributed to the study design and the drafting of the manuscript. Where possible all authors read and approved the final manuscript.

## Pre-publication history

The pre-publication history for this paper can be accessed here:

http://www.biomedcentral.com/1472-6947/12/122/prepub
